# A Novel Effective Formulation of Bioactive Compounds for Wound Healing: Preparation, *In Vivo* Characterization, and Comparison of Various Postbiotics Cold Creams in a Rat Model

**DOI:** 10.1155/2021/8577116

**Published:** 2021-12-07

**Authors:** Nasim Golkar, Yousef Ashoori, Reza Heidari, Navid Omidifar, Seyedeh Narjes Abootalebi, Milad Mohkam, Ahmad Gholami

**Affiliations:** ^1^Department of Pharmaceutics, School of Pharmacy, Shiraz University of Medical Sciences, Shiraz, Iran; ^2^Pharmaceutical Sciences Research Center, Shiraz University of Medical Sciences, Shiraz, Iran; ^3^Biotechnology Research Center, Shiraz University of Medical Sciences, Shiraz, Iran; ^4^Department of Pharmaceutical Biotechnology, School of Pharmacy, Shiraz University of Medical Sciences, Shiraz, Iran; ^5^Student Research Committee, Shiraz University of Medical Sciences, Shiraz, Iran; ^6^Department of Pathology, School of Medicine, Shiraz University of Medical Sciences, Shiraz, Iran; ^7^Division of Intensive Care Unit, Department of Pediatrics, School of Medicine, Shiraz University of Medical Sciences, Shiraz, Iran; ^8^Allergy Research Center, Shiraz University of Medical Sciences, Shiraz, Iran

## Abstract

The wound is a break in the integrity of the skin produced by injury, illness, or operation. Wound healing is an essential dynamic biological/physiological process that occurs in response to tissue damage. The huge health, economic, and social effects of wounds on patients and societies necessitate the research to find novel potential therapeutic agents in order to promote wound healing. Postbiotics, the newest member of the biotics family, are valuable functional bioactive substances produced by probiotics through their metabolic activity, which have several beneficial properties, including immunomodulatory, anti-inflammatory, antimicrobial, and angiogenesis characteristics, resulting in acceleration of wound healing. In the current study, three topical cold cream formulations containing postbiotics obtained from *Lactobacillus fermentum*, *Lactobacillus reuteri,* or *Bacillus subtilis* sp*. natto* probiotic strains were prepared. The effectiveness and wound healing activity of the developed postbiotics cold cream formulations were investigated compared to cold cream without postbiotics and no treatment via wound closure investigation, hydroxyproline content assay, and histological assessment in 25 Sprague Dawley rats divided into five groups. Interestingly, analysis of the results revealed that all three formulations containing postbiotics significantly accelerated the wound healing process. However, in general, the *Bacillus subtilis natto* cold cream manifested a better wound healing property. The pleasing wound healing characteristics of the topical postbiotics cold creams through the *in vivo* experiment suggest that formulations containing postbiotics can be considered as a promising nominee for wound healing approaches.

## 1. Introduction

A term wound is defined as a cut or break in the integrity of the skin produced by injury, illness, or operation [1–3]. Wounds can happen due to a disease or as a consequence of an accidental or intentional reason [4, 5]. The principal function expressed for the skin is to provide a protective barrier for the body against the surrounding environment [6]. Loss of skin unity provides an appropriate context for various microorganisms to contaminate the wound surface [2, 7]. As intact skin is vital to protect the body against the environment, regenerative mechanisms (healing) need to be initiated and progressed to resolve the existing defect [1, 8].

Cutaneous wound healing is a dynamic complex biological phenomenon that commences following tissue injury. The critical goal of wound healing is inhibition against infections, restoring skin tissue function and strength [1, 4, 9]. A wound as a tissue injury stimulates a regulated and coordinated response, and the wound healing process is attained through 4 principal precise physiological phases of homeostasis, inflammation, proliferation, and remodeling [1, 2, 4, 10]. In the first phase of the healing cascade that is hemostasis, platelets are activated, and growth factors and cytokines along with other substances are secreted, which in turn stimulates the mechanisms of tissue repairing resulting in inflammation, proliferation, angiogenesis, deposition of extracellular matrix (ECM), and finally tissue remodeling [2, 9, 11, 12].

Many items can interfere with the wound healing process leading to delayed or impaired wound healing, which represents a significant cause of patient morbidity, mortality, and poor cosmetic consequence [2, 4, 13, 14]. Furthermore, wounds' health, economic burden, and social effects are among other substantial problems requiring special consideration [1, 2, 15, 16]. Wounds represent a major worldwide challenge for patients and their families, health institutions, and caregivers [17, 18]. Regarding the economic aspect, the annual cost of wound-related complications in the United States alone is more than 1 billion dollars [4, 13]. Consequently, higher levels of attention and research are needed to investigate the novel potential therapeutic agents that can fulfill one of the primary goals of wound treatments, speeding the process of wound healing [1, 2, 4, 6].

Probiotics are live microorganisms used in the appropriate amounts, which positively impact host health [19–23]. The advantages of probiotic bacteria for wound healing have been proposed extensively via induction of the immune system, decrease of inflammation, angiogenesis, and antimicrobial properties [2, 3, 20, 24–29]. Recently, there has been an increasing interest in the newest member of the biotics family, postbiotics [19, 30]. Postbiotics are functional bioactive substances produced through the metabolic activity of the probiotics during fermentation, which directly and/or indirectly exert beneficial effects on the host cells. Postbiotics can include many components such as metabolites, cell fractions, cell lysates, short-chain fatty acids (SCFAs), extracellular polysaccharides (EPS), teichoic acid, proteins, and peptidoglycan-derived muropeptides as well as pili type structures [19, 23, 31]. Although postbiotics do not have live microorganisms, they show a beneficial impact on host health by similar mechanisms that are features of probiotics, diminishing the possible risks accompanying their intake. Therefore, postbiotics seem to be safe due to the lack of any possible side effects that may be introduced for live microorganisms while preserving similar effectiveness like probiotics [30, 31].

Topical drug delivery is applying a formulation to the skin tissue to treat a cutaneous disorder or improvement of the cutaneous appearance of a disease. Creams, topical semisolid preparations, are among the widely used therapeutic or cosmetic preparations in many skin conditions. They can be utilized to any part of the body and by all age groups easily and efficiently [32].

Postbiotics' topical application can be considered as a novel therapeutic approach in wound research to accelerate the healing process. The present study aimed to examine the impact of postbiotics formulations (prepared in the form of cream) on the wound healing process. Accordingly, three novel formulations of cold cream containing postbiotics were developed to enhance the wound healing process possibly. The efficacy of the prepared postbiotics creams was investigated through *in vivo* assessments, including wound sizes, wound healing percentages, hydroxyproline content assay, and histopathological evaluation in a rat model.

## 2. Materials and Methods

### 2.1. Materials

Tryptic soy broth (TSB), De Man, Rogosa & Sharpe (MRS) broth, and yeast extract were purchased from Himedia (India). Soy-peptone was from Quelab (Canada), and magnesium sulfate, potassium hydrogen phosphate, maltose, and glucose were obtained from Merck (Germany). N-chloro tosylamide (chloramine-T), p-dimethyl amino benzaldehyde, pure L-hydroxyproline, perchloric acid, and n-propanol were also purchased from Merck (Germany). All the other reagents, solvents, and salts used for buffer solution preparations were of analytical grade and acquired from Merck (Germany).

### 2.2. Preparation of Postbiotics

The three utilized probiotic bacteria in the present study were *Bacillus subtilis* sp*. natto* (*B.S.* natto, ATCC 15245), *Lactobacillus reuteri* (*L. reuteri*, ATCC 23272), and *Lactobacillus fermentum* (*L. fermentum*, ATCC 9338) strains.

The *L. reuteri* and *L. fermentum* bacteria were first cultured consuming MRS broth medium at 37°C for 48 h under microaerophilic conditions until the achievement of the stationary phase. *B.S. natto* strain was cultured utilizing soy-peptone (10 g), magnesium sulfate (1 g), potassium hydrogen phosphate (2 g), maltose (20 g), glucose (2 g), and yeast extract (10 g) and incubated at 37°C for 48 h. The pH was adjusted to 7.2. The inoculum of each strain was prepared at an approximate density of 1 × 10^8^ to 1 × 10^9^ CFU/mL. The number of viable bacteria was measured via plate counts utilizing MRS agar, and the bacteria were then harvested using Eppendorf 5810R centrifuge (Germany) at 4000 rpm for 20 min at 4°C. Filtration of the supernatants was performed using a membrane filter of 0.2 *μ*m to omit the remaining bacteria and other remains. The filtered supernatants were lyophilized using an Alpha 1-2LD Plus lyophilizer (Martin Christ, Germany) and stored at −20°C. There was not any evidence of lactobacilli growth in bacterial counting of MRS agar plates. The existence of lipopolysaccharide in L.S. had been investigated via a diagnostic kit from Cambrex Corporation (East Rutherford, NJ).

### 2.3. Preparation of Cold Cream

The cold cream is based on water in oil (w/o) emulsion. Beeswax (15% w/w) and liquid paraffin (45% w/w) were taken in a beaker and heated up to 70°C using a water bath to prepare the oily phase. As an aqueous phase, borax (1% w/w) was dissolved in water (qs to 100) in another beaker and heated to 75°C. The aqueous phase was slowly added to the oily phase with continuous stirring at −4°C until a cream consistency was obtained (20 g cream). The prepared cream was packed in a suitable container and stored in a cool and dry place for further use.

### 2.4. Preparation of Postbiotics Cold Creams

Each lyophilized postbiotic at the amount of one milligram was added to 10 grams of the prepared cold cream and mixed for 5 min at room temperature to develop three different postbiotics cold cream formulations as follows:

Formulation 1: *Lactobacillus fermentum* postbiotic cold cream; Formulation 2: *Lactobacillus reuteri* postbiotic cold cream; and Formulation 3: *Bacillus subtilis* sp*. natto* postbiotic cold cream.

### 2.5. Animal Study

#### 2.5.1. Study Design

Twenty-five mature Sprague Dawley rats were purchased from the Center of Comparative and Experimental Medicine, Shiraz University of Medical Science, possessing a bodyweight in the range of 200 to 300 g. The rats were housed in a standard cage wherein an ordinary standard rodent's pellet chow diet (RoyanFeed®, Isfahan, Iran) and tap water were available. Before initiation of the experiment, the animals were maintained in the new provided situation at the temperature of 25°C ± 1°C and 12 h/12 h light/dark photo schedule along with relative humidity of 40% ± 10% for 15 days to remove any impacts of stress on them. During the acclimatization period, the rats were examined for their health by a veterinarian. After this period, the rats were equally allotted into five groups (Table 1) containing five rats in each.

#### 2.5.2. Wound Creation

Each rat was then anesthetized using an anesthesia mixture including ketamine and xylazine at the amount of 80 mg/kg and 10 mg/kg, respectively, followed by shaving the needed area to remove hair, cutting a skin layer, and creating an excision wound of 226 mm^2^ square with 2 mm depth.

#### 2.5.3. Wound Healing Activity Measurement

Topical postbiotics formulations, prepared at a concentration of 1% W/W postbiotic in cold cream, were administered to the created wounds once a day repeated for two weeks (14 days). The rats receiving nothing and the rats receiving the formulation without any postbiotics were considered as control groups. The diameters of the excised wounds in each rat group were recorded on days of 1 to 14. The following equation calculated wound healing percentage:

(1) $$\begin{array}{c} \text{wound Healing}\%=\left(1-\frac{\text{WSt}}{\text{WS0}}\right)\times 100, \end{array}$$

where WS_t_ and WS_0_ are the wound size on a specific day and day 0, respectively.

#### 2.5.4. Hydroxyproline Assay

Hydroxyproline (HP) content was measured as an index of collagenesis according to the method first described by Woessner [33] with further modifications [34]. Briefly, at the end of Day 14, a piece of skin tissue from the healed wound zone was collected and analyzed for the hydroxyproline content. Each skin sample was dried at 60°C to obtain and record a constant dry weight. Skin tissue homogenate at a concentration of 20% w/v was prepared in phosphate-buffered saline (PBS, pH = 7.4), and 500 *μ*l of the mixture was hydrolyzed in 1 ml of 6 N HCl (hydrochloric acid). Following incubation in a sealed tube for 8 h at 120°C, the hydrolysate (25 *μ*l) was mixed with 25 *μ*l of citrate-acetate buffer (pH = 6) to be neutralized and was subjected to 500 *μ*l of chloramines-t-solution (56 Mm). The resulting mixture was allowed to remain at room temperature for 20 min. Afterward, 500 *μ*l of Ehrlich's reagent (15 g of p-dimethyl amino benzaldehyde in 2 : 1 v/v n-propanol/perchloric acid) was added, followed by incubation for 15 min at 65°C. Perchloric acid and Ehrlich's reagent were used as reaction terminators and color developers, respectively. After cooling, the absorbance values of the developed pink color were measured at the wavelength of 550 nm using a spectrophotometer (Ultrospec 2000®UV, Pharmacia Biotech, Sweden). The experiment was performed similarly for all the rat groups receiving different formulations.

#### 2.5.5. Wound Histopathological Evaluation and Scoring

The rats were first anesthetized and then sacrificed by spinal cord injury. Full-thickness wound skin tissues (each wound at dimensions of 3.5 cm × 1.2 cm) were detached. After preparation of the paraffin-embedded sections, they (each with 2 mm thickness) were cut vertically to the width of the skin surface followed by staining with a combination of two histological stains called hematoxylin-eosin [35–38]. The hematoxylin precisely stains cell nuclei, while eosin stains the extracellular matrix and cytoplasmic components. Then, the histological alterations of skin tissue through investigation of different phases of epithelialization, fibrosis, inflammation, and granulation were evaluated for all the samples. To quantify the wound healing process, degrees of epithelialization, fibrosis, inflammation, and granulation were blindly scored by a professional pathologist, as presented in Table 2.

### 2.6. Statistical Analysis

Data analysis was performed using GraphPad software version 8 (v8.4.0, GraphPad Software Inc., San Diego, CA). Quantitative variables were expressed as mean ± standard deviation (SD). The comparisons were carried out by analysis of variance (ANOVA) with Tukey's comparison post hoc test. Statistical significance was defined as *P* values less than 0.05 (*P* < 0.05). Scores of histopathological skin changes are presented as median and quartiles, and the Kruskal-Wallis, followed by the Mann–Whitney *U* test, was employed to analyze the skin tissue histopathological changes.

## 3. Results

### 3.1. Wound Healing Activity Measurement

**Figure 1:**
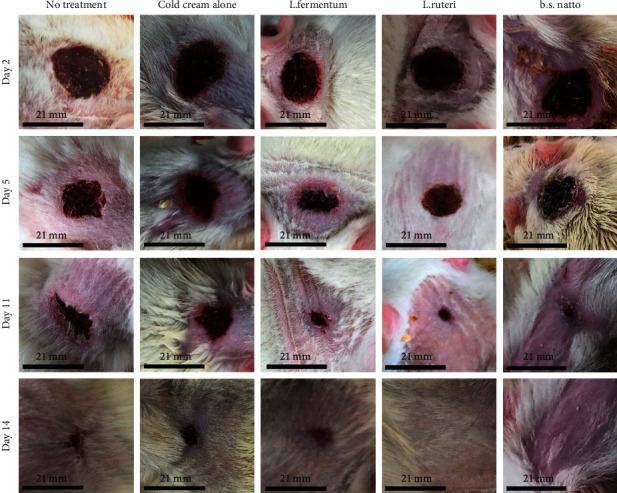
Qualitative trend of wound healing process of different rat groups receiving no treatment (control), cold cream alone, *Lactobacillus fermentum* postbiotic cold cream, *Lactobacillus Reuteri* postbiotic cold cream, or *Bacillus Subtilis* sp*. natto* postbiotic cold cream. The photos were taken at Day 2, Day 5, Day 11, and Day 14 of treatment.

**Figure 2:**
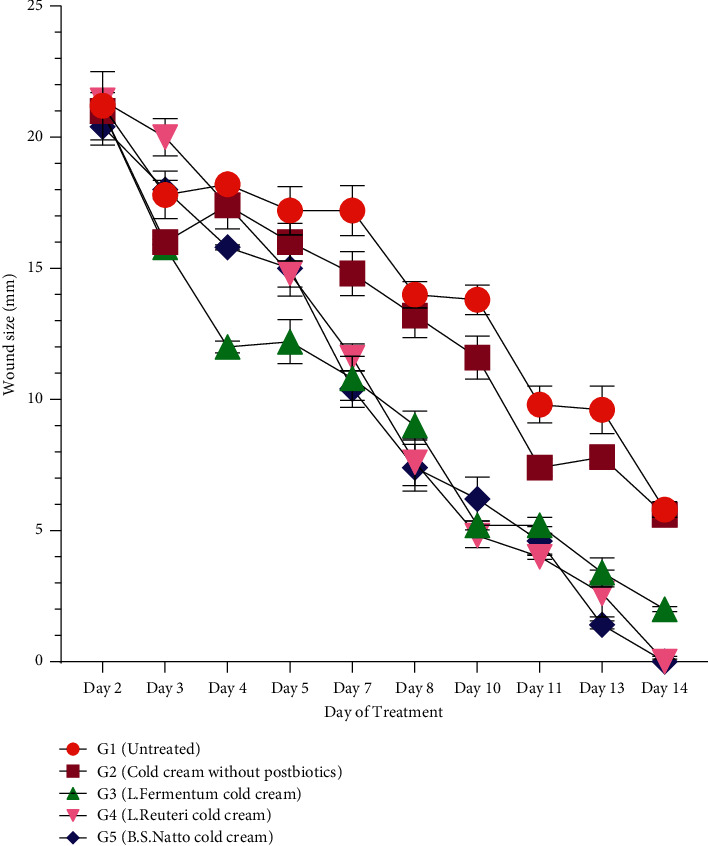
Wound sizes (mm) following treatment of different rat groups (G1 to G5) with various formulations. Data are expressed as mean ± SD (standard deviation).

**Figure 3:**
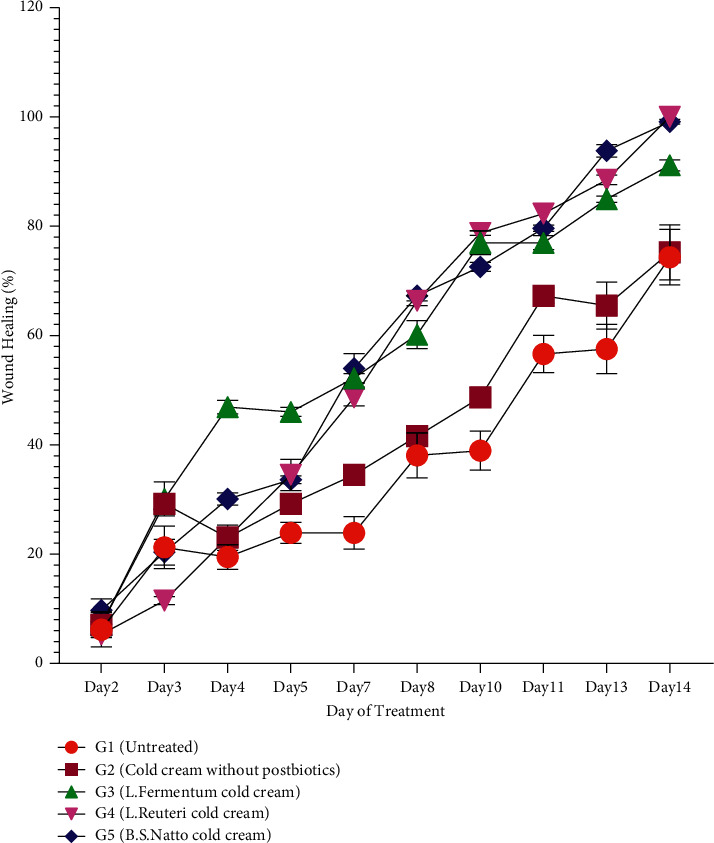
Wound healing percentages following treatment of different rat groups (G1 to G5) with various formulations. Data are expressed as mean ± SD (standard deviation).

Various rat groups were topically treated with three different postbiotics cold creams (G3, G4, and G5), cold cream without postbiotic (G2), and no treatment (G1) to evaluate their wound healing process as well as compare their abilities to improve the rate of wound healing through measurement of wound sizes and wound healing percentages in different days of treatment. The qualitative trend of wound healing in treated rat groups was demonstrated in Figure 1. As it is clear, the process of wound healing was typically initiated and progressed in all groups. However, the rate of wound healing is more in all three groups treated with postbiotics cold cream formulations (G3, G4, and G5) than in the group with no treatment (G1) and the group treated with cold cream alone (G2). The quantitative wound healing trends of all rat groups obtained from wound size against days of treatment and wound healing percentage against days of treatment were illustrated in Figures 2 and 3, respectively. Moreover, the healing percentages were summarized in Table 3.

The wound size was 22.5 ± 1.00 mm on the first day (Day 0) for each rat in all the groups, equivalent to 0% wound healing percentage. The trend of wound sizes (Figure 2) and wound healing percentages (Figure 3) decreased and increased, respectively, in all rat groups. Wound sizes decreased significantly (Figure 2, *P* < 0.0001), and wound healing percentages increased significantly (Figure 3, *P* < 0.0001) from Day 2 to Day 14 in all five rat groups of G1 to G5. There was not any significant difference (*P* > 0.05) between the group receiving no treatment (G1) and the group receiving cold cream without postbiotics (G2) during almost all days of the treatment period (from Day 0 to Day 14) regarding wound size (Figure 2) and wound healing percentage (Figure 3). From Day 4 to the end of the treatment (Day 14), all the three postbiotics cold creams (G3, G4, and G5) showed smaller wound sizes (Figure 2) and higher wound healing percentages (Figure 3) significantly in comparison with the untreated group (G1) and the group treated with cold cream without postbiotics (G2) (*P* < 0.0001). The administration of cold cream alone did not significantly enhance the wound healing process compared to the treatment with postbiotics cold creams (*P* > 0.0001). The wound sizes were the smallest (Figure 2, *P* < 0.001), and the wound healing percentages were the highest (Figure 3, *P* < 0.001) significantly from Day 4 to Day 14 of treatment in the groups receiving the *L. reuteri* cold cream (G4) and *B.S.* sp. *natto* cold cream (G5) followed by *L. fermentum* cold cream (G3). The wound healing process in two groups receiving *L. reuteri* cold cream (G4) and *B.S.* sp. *natto* cold cream (G5) was completed by Day 14 in which the wound sizes obtained were 0 (Figure 2), and the healing percentages reached 100% (Figure 3). The wound size and wound healing percentage in the group receiving *L. fermentum* cold cream (G3) were measured 2.000 ± 0.100 (Figure 2) and 91.150 ± 1.000 (Figure 3 and Table 3) by Day 14. The healing process in the group receiving no treatment (G1) and the group receiving cold cream without postbiotics (G2) was not completed at the end of the experiment (Day 14) (Figures 2 and 3 and Table 3) with the respective wound sizes of 5.800 ± 0.250 and 5.600 ± 0.210 and the respective wound healing percentages of 74.336 ± 4.000 and 75.221 ± 3.030.

### 3.2. Hydroxyproline Assay

**Figure 4:**
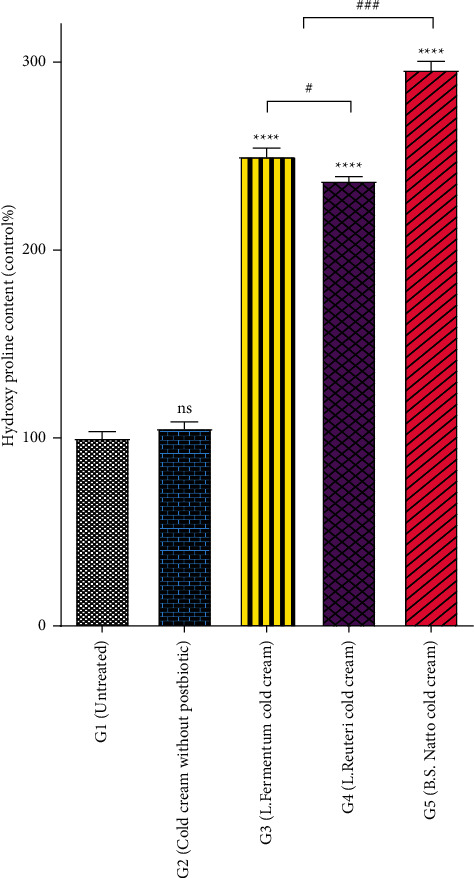
Determination of wound hydroxyproline production as an indicator of collagen levels measured at the end of Day 14 following administration of three different postbiotics cold creams (*Lactobacillus fermentum* postbiotic cold cream, *Lactobacillus Reuteri* postbiotic cold cream, or *Bacillus subtilis* sp. *natto* postbiotic cold cream), cold cream without postbiotics, and no treatment (control) on the excised wounds in rat model. Result values are expressed as means ± standard deviation. ^*∗∗∗∗*^*P* value < 0.0001, ^*∗∗∗*^*P* value < 0.001, and ^*∗*^*P* value < 0.05; ns denotes not significant compared to untreated wound tissue. ^###^*P* value < 0.001 and ^#^*P* value < 0.05.

Hydroxyproline is a basic component of collagen, and its measurement can be used as a biomarker for collagenesis in skin tissue [39]. The calculated hydroxyproline content related to various rat groups receiving various postbiotic cold creams, cold cream without postbiotic, and no treatment is shown in Figure 4. The hydroxyproline content in wound tissue of the groups treated with each of three postbiotics cold creams (G3 to G5) significantly increased (*P* < 0.0001) in comparison with the wound tissues of the groups receiving no treatment (G1) or cold cream without postbiotic (G2) (Figure 4). Interestingly, all three postbiotics cold creams showed a significantly higher amount of hydroxyproline than the untreated and the cold cream alone groups (*P* < 0.0001). Among three postbiotics cold creams, the produced hydroxyproline was the highest for the group treated with *B.S. natto* cold cream (G5; 296 ± 4, *P* < 0.0001). Moreover, the result of comparing the other two postbiotics formulations demonstrated that the hydroxyproline content of the group treated with *L. fermentum* cold cream (G3) was significantly higher (252 ± 4, *P* < 0.05) than the group treated with *L. reuteri* cold cream (G4) (236 ± 3). The hydroxyproline content in the untreated group (G1) and the group treated with cold cream alone (G2) was the least of all (*P* < 0.0001) without any significant difference between them (*P* > 0.05).

### 3.3. Wound Histopathological Evaluation

**Figure 5:**
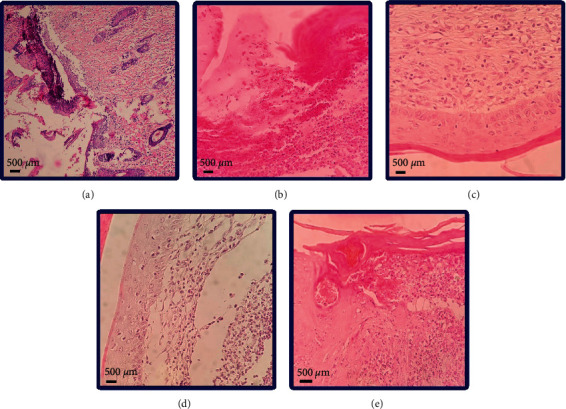
Skin tissue histopathological assessment of different rat groups at the end of Day 14 through hematoxylin-eosin staining. (a) Rat group receiving no treatment (control). (b) Rat group receiving cold cream alone. (c) Rat group receiving *Lactobacillus fermentum* postbiotic cold cream. (d) Rat group receiving *Lactobacillus Reuteri* postbiotic cold cream. (e) Rat group receiving *Bacillus subtilis* sp*. natto* postbiotic cold cream.

The histological assessment at the end of the treatment (Day 14) with various postbiotics cold creams, cold cream without postbiotic, and no treatment was performed to determine the histological characterizations of wound healing. Skin tissue histopathological changes are demonstrated in Figure 5, and the scores by an expert given to different processes of epithelization, inflammation, granulation, and fibrosis are presented in Table 4.

As illustrated in Figure 5 and expressed in Table 4, the epithelialization process was complete in the groups of rats receiving *L. reuteri* cold cream (G4) and *B.S. natto* cold cream (G5), while the epithelization in groups receiving *L. fermentum* cold cream (G3), cold cream without postbiotics (G2), and no treatment (G1) was incomplete (super), incomplete (deep), and incomplete, respectively.

According to the inflammation process (Figure 5 and Table 4), the groups treated with cold cream without postbiotics (G2) and the group receiving no treatment (G1) as well as *L. fermentum* cold cream (G3) possessed the highest degree of inflammation (defined as moderate inflammation) in comparison to the other groups. The skin inflammation differed among the groups treated with various postbiotics cold creams (G3, G4, and G5). The group treated with *B.S. natto* cold cream (G5) showed a mild degree of inflammation. However, treatment of the rats with *L. reuteri* cold cream (G4) resulted in no inflammation.

The degree of granulation (Figure 5 and Table 4) was moderate for the groups treated with cold cream without postbiotics (G2) and the group receiving no treatment (G1), which was the highest among the studied groups, followed by the group receiving *L. fermentum* cold cream (G3) which was defined as mild granulation. The groups were treated with *L. reuteri* cold cream (G4), and *B.S. natto* cold cream (G5) did not demonstrate any histological alterations regarding granulation.

Although the fibrosis process was detected in all the studied groups, it is significantly higher in the group receiving no treatment (defined as moderate fibrosis). Mild fibrosis was observed in the other four groups (G2, G3, G4, and G5) (Figure 5 and Table 4).

## 4. Discussion

The wound is a break or cut in the skin, and wound healing is a dynamic, complex physiological reaction that initiates following skin injuries. The enormous health, social, and economic challenges associated with wounds lead to finding novel therapeutic agents that can enhance the wound healing process. Postbiotics, functional bioactive substances produced by probiotics, have recently attracted a great deal of interest due to many beneficial characteristics. In the present study, three new formulations (postbiotics cold creams) were developed to study their efficacy in the wound healing process via *in vivo* experiment in a rat model.

According to the wound sizes (Figure 2) and wound healing percentages (Figure 3 and Table 3), at the end of a 14-day treatment, wound sizes decreased (Figure 2), and wound healing % increased significantly (Figure 3 and Table 3) by increasing day of treatment in all five groups (G1 to G5) but in different rates which demonstrates that the process of wound healing was initiated and progressed regardless of the healing rate. However, wound sizes were smaller and wound healing percentages were higher from Day 4 to Day 14 in all three groups which received postbiotics cold creams (G3, G4, and G5) than the group with no treatment (G1) and the group treated with cold cream (G2), which indicates the higher rates of wound healing in all the three postbiotics groups than the controls. Administration of cold cream without postbiotics (G2) did not increase the wound healing process significantly (which was the same as no treatment group) (Figures 2 and 3 and Table 3) in comparison to the treatment with postbiotics cold creams (G3 to G5), which confirms that the higher activity of wound healing factors was associated with the postbiotics substances in these formulations. The smallest wound sizes (Figure 2) and the highest wound healing percentages (Figure 3) in Day 4 to Day 14 of treatment were allocated to the groups receiving the *L. reuteri* cold cream (G4) and *B.S.* sp. *natto* cold cream (G5), followed by *L. fermentum* cold cream (G3). Moreover, the wound healing process in groups receiving *L. reuteri* cold cream (G4) and *B.S.* sp. *natto* cold cream (G5) was completed by Day 14 (Figures 2 and 3 and Table 3). The wound healing process in the group receiving *L. fermentum* cold cream (G3) reached near completion (90%) by Day 14 (Figures 2 and 3 and Table 3), while the healing process in the group receiving no treatment (G1) and the group receiving cold cream without postbiotics (G2) was not completed even at the end of the treatment (Day 14) (Figures 2 and 3 and Table 3). It reveals that the *L. reuteri* and *B.S.* sp. *natto* postbiotics could accelerate the healing process more than the others regarding wound sizes and wound healing percentages, followed by *L. fermentum*. Previous studies demonstrated that the administration of *L. reuteri* and could significantly enhance the wound healing process through exerting various properties such as anti-inflammatory and antipathogenic ones [40–42]. In consistent with the findings of this study, some studies showed the postbiotics as probiotic metabolites can effectively boost the wound healing process. However, the amount of their efficacy may depend on bacterial strain, amount of administration, and other factors.

Collagen is the major structural component of granulation tissue, strengthening the extracellular matrix. It was demonstrated that a collagen sponge improves the formation of the connective tissue and increases the vascularization related to the repaired tissue. As a result, collagen is effectively able to increase the healing process [43, 44]. The hydroxyproline amount of wound tissues is assessed to estimate the amount of produced collagen in the wound healing process. Because the amino acid proline is a crucial component of the collagen fiber, hydroxyproline can be considered an index of collagenesis. Accordingly, the higher amount of hydroxyproline positively indicates the higher progression of wound healing [17, 39, 45]. Therefore, the higher amount of hydroxyproline in postbiotics formulations (Figure 4) suggests that all the 3 postbiotics cold creams, regardless of their bacterial source, can enhance the wound healing process. Among three postbiotics cold creams, the produced hydroxyproline was the highest for the group treated with *B.S. natto* cold cream (G5) (Figure 4), suggesting its highest wound healing as a consequence of the highest collagenesis. Previous studies also have demonstrated that their studied formulations showed higher collagen amount and therefore could promote the wound healing process [46–49].

The histological evaluation can reveal important facts about the process of wound healing. Wound healing consists of several organized mechanisms and is affected by different factors. Epithelialization, a major component of wound healing, happens in the proliferative phase and is used as a defining parameter for healing success. In the absence of reepithelialization, a wound is not able to be healed [50]. Epithelialization is a process in which epithelial cells migrate upwards and renovate the wounded zone. Skin stem cells located in the epidermis contribute to the reepithelialization when the skin is injured. The epithelialization process is activated by the inflammatory signal. After that, the keratinocyte migrates and differentiates to close the skin defect [51]. Epithelialization was complete in the rat groups receiving *L. reuteri* cold cream (G4) and *B.S. natto* cold cream (G5), while the epithelization observed in other groups (G1, G2, and G3) was incomplete, demonstrating that *L. reuteri* and *B.S. natto* resulted in probable higher wound healing as the accelerated epithelization means promoted wound healing. Previous studies have also shown that accelerated epithelization led to boosting the wound healing process [25, 42, 47, 48]. At the beginning of injury, the inflammation is activated to stimulate the wound healing process. In the inflammation phase, the mediators contribute to the infiltration of immune cells into the inflammation site. However, the apoptosis of the immune cells and clearance of the apoptotic cells by macrophages results in the end of inflammation, and by a decrease of the inflammation, the wound healing process initiates and progresses [52]. According to the inflammation process, the groups treated with cold cream without postbiotics (G2) and the groups receiving no treatment (G1) and *L. fermentum* cold cream (G3) had the highest degree of inflammation among all five groups (Figure 5 and Table 3) which propose that these three groups possessed lower wound healing than *L. reuteri* (G4) and *B.S. natto* (G5). Besides, the inflammation was the weakest for *L. reuteri* (G4) and *B.S. natto* (G5) postbiotics (Figure 5 and Table 3) with the observation of no inflammation and mild inflammation, respectively, demonstrating better wound healing in these two postbiotics groups. Previous studies have shown similar results regarding epithelization in the wound healing process [25, 42, 46, 47, 53]. Regarding granulation, groups treated with *L. reuteri* cold cream (G4) and *B.S. natto* cold cream (G5) did not show any histological changes (Figure 5 and Table 3), and the group receiving *L. fermentum* cold cream (G3) showed mild granulation (Figure 5 and Table 4), while observed granulation for the groups treated with cold cream without postbiotics (G2) and the group receiving no treatment (G1) was moderate. The results suggest that the healing process is better in *L. reuteri* (G4) and *B.S. natto* postbiotics followed by *L. fermentum*. The fibrosis process obtained was mild in all groups (G2 to G5) except for the no-treatment group (G1), which resulted in moderate fibrosis (Figure 5 and Table 4).

In the present study, the conventional cream, cold cream, was used, which itself did not show any efficacy towards the promotion of wound healing. Consequently, the positive effect of postbiotics cold creams on wound healing was related to the nature of postbiotics. The efficacy of postbiotics in wound healing may be due to the metabolites [30]. Postbiotics include many substances such as cell fractions, cell lysates, short-chain fatty acids (SCFAs), extracellular polysaccharides (EPS), teichoic acid, and proteins [19]. These secreted metabolites can stimulate proteoglycans deposition, angiogenesis, reduction of inflammation through reduction of the expression of proinflammatory cytokines, and secretion of growth factors like EGF [25, 26, 40, 54–59]. All these effects can be responsible for the good efficacy of postbiotics in the improvement of wound healing. In the past, researchers thought that probiotics were only advantageous in gastrointestinal diseases [60]. However, extensive research leads to understanding their importance in daily life and many disorders [24, 27, 61]. There are some studies relating to the effects of probiotics on wound healing [40, 42, 62, 63], but there are few studies evaluating postbiotics, particularly for wound healing because its recognition does not go far in the past, and it is regarded as a new member of biotics family. Various novel formulations such as hydrogels [46, 47, 53, 64], chitosan nanogels [65], microspheres [64, 66], nanoparticles [64, 67–69], liposomes [64], asymmetric membranes [70], and a lot more have been studied in wound healing. Interestingly, it might be possible to use these novel formulations in combination with postbiotics biocompounds in order to enjoy the probable synergic efficacy in the enhancement of wound healing. Moreover, pH of the wounds is acidic. pH of the postbiotics is also acidic, which can be compatible with wound situations. Therefore, this matter can open new frontiers for developing smart or targeted formulations of postbiotics such as pH-sensitive ones in wound healing. Accordingly, this novel postbiotics formulation may open a new horizon for the treatment of wound healing in the future.

## 5. Conclusion

The results revealed that wound treatment with formulations of postbiotics cold creams in a rat model accelerated a wound healing rate in comparison to no-treatment and cold cream without postbiotics-treated rat groups. According to the wound sizes and wound healing percentages, *B.S. natto* and *L. reuteri* were the best. Regarding hydroxyproline content, *B.S. natto* produced the highest amount of hydroxyproline, and histological characterization manifested the best wound healing for *L. reuteri* and *B.S. natto*. Generally, the results propose that the prepared novel postbiotics formulation can be considered a supporting wound healing therapy.

## Figures and Tables

**Table 1:** Different rat groups treated with various topical postbiotics formulations.

Rat groups	The topical formulation administered to the rat group
**Group 1**	No treatment (control)
**Group 2**	Cold cream without postbiotics
**Group 3**	Formulation 1 (*Lactobacillus fermentum* postbiotic cold cream)
**Group 4**	Formulation 2 (*Lactobacillus reuteri* postbiotic cold cream)
**Group 5**	Formulation 3 (*Bacillus subtilis* sp*. natto* postbiotic cold cream)

**Table 2:** Scores of the changes in histopathological features of the skin.

Score	Changes in histopathological features of skin tissue
**−**	There is no apparent change
**++**	Mild changes
**++**	Moderate changes
**+++**	Severe changes

**Table 3:** Wound healing percentages in various days of a 14-day treatment of five rat groups with three postbiotic cold cream formulations, cold cream formulation alone, and no treatment (control).

Rat groups	No treatment (control)	Cold cream alone	L. *fermentum* cold cream	L. *reuteri* cold cream	*B. S. natto* cold cream
Day					
**2**	6.195 ± 0.330	7.800 ± 0.350	7.080 ± 0.600	5.310 ± 0.300	9.734 ± 0.400
**3**	21.239 ± 1.000	29.203 ± 1.000	30.088 ± 0.900	11.504 ± 0.710	20.354 ± 0.350
**4**	19.469 ± 0.890	23.009 ± 0.890	46.902 ± 1.210	23.009 ± 1.300	30.088 ± 1.100
**5**	23.894 ± 0.710	29.203 ± 0.710	46.017 ± 0.840	34.513 ± 2.000	33.628 ± 0.710
**7**	24.1 ± 0.840	34.513 ± 0.840	52.212 ± 0.840	48.672 ± 1.520	53.982 ± 2.700
**8**	38.053 ± 0.800	41.593 ± 0.800	60.177 ± 2.550	66.372 ± 0.890	67.256 ± 0.890
**10**	38.938 ± 1.500	48.672 ± 1.820	76.991 ± 2.170	78.761 ± 0.450	72.566 ± 0.840
**11**	56.637 ± 1.340	67.257 ± 1.340	76.995 ± 1.300	82.301 ± 0.100	79.646 ± 0.550
**13**	57.522 ± 3.000	65.487 ± 3.320	84.956 ± 0.550	88.495 ± 0.890	93.805 ± 1.150
**14**	74.336 ± 4.000	75.221 ± 3.030	91.150 ± 1.000	100.00 ± 0.010	100.00 ± 0.850

**Table 4:** Skin histopathological changes at the end of Day 14 in different rat groups following 14-day administration of various formulations on wound tissues.

	No treatment (control)	Cold cream	*L. fermentum* cold cream	*L. reuteri* cold cream	*B.S. natto* cold cream
**Epithelization**	Incomplete	Incomplete (deep)	Incomplete (super)	Complete	Complete
**Inflammation**	++	++	++	−^*∗*^	+^*∗*^
**Granulation**	++	++	+^*∗*^	−^*∗*^	−^*∗*^
**Fibrosis**	++	+^*∗*^	+^*∗*^	+^*∗*^	+^*∗*^

− means no significant change (*P* > 0.05); + and ++ mean mild (*P* < 0.05) and moderate (*P* < 0.001) histopathological significant changes, respectively. ^*∗*^Significant histopathological changes in comparison to the no-treatment group (control) (*P* < 0.05).

## Data Availability

All the data generated or analyzed during this study are included in this article.
